# Could peripheral 5-HT level be used as a biomarker for depression diagnosis and treatment? A narrative minireview

**DOI:** 10.3389/fphar.2023.1149511

**Published:** 2023-03-07

**Authors:** Canye Li, Qiming Cai, Zuanjun Su, Zhicong Chen, Jinming Cao, Feng Xu

**Affiliations:** ^1^ Fengxian Hospital, Southern Medical University, Shanghai, China; ^2^ Sixth People’s Hospital South Campus, Shanghai Jiaotong University, Shanghai, China

**Keywords:** 5-HT, peripheral, depression, biomarker, plasma, platelet

## Abstract

The serotonin hypothesis of depression is still influential, but the relationship between peripheral 5-HT levels and depression is still unknown. This review aimed to verify whether peripheral 5-HT levels could be used as a biomarker for depression diagnosis and treatment. PubMed and EMBASE were searched using terms appropriate to the area of research. Articles from 1957 to 2022 in the following terms were identified: depression, 5-HT, serotonin and peripheral (serum, plasma, blood platelets). 33 studies were included: seven clinical trials about periphery 5-HT levels in depressive patients compared to normal subjects, 15 clinical trials about changes of peripheral 5-HT levels in patients with depression after drug treatment and 11 animal experiments about peripheral 5-HT levels in animal models of depression. Peripheral 5-HT levels presented three different outcomes before and after antidepressant treatments: increased, decreased and no significant change. In conclusion, changes in peripheral 5-HT levels did not show consistent results among these studies. Peripheral 5-HT level could not be used as a biomarker both for depression diagnosis and for antidepressant efficacy evaluation.

## Introduction

Depression is a common mental disorder. Patients with depression tend to exhibit slowness of thought, agitation and sustained low mood. Those with major depressive disorder (MDD) may suffer from a range of other symptoms such as psychomotor disorders and cognitive impairment, even suicide (Kandola et al., 2019). According to a World Health Organization report, approximately 280 million people (about 3.8% of the total population) are suffering from depression worldwide. More than 75% of depression patients in developing countries do not receive proper diagnosis and treatment (Herrman et al., 2022).

Decades of studies on the pathological mechanisms of depression have not yet led to a complete elucidation. The development of depression is usually considered to be related to numerous factors including genetic, environmental, immune, endocrine and neurogenesis (Jesulola et al., 2018), and many pathogenetic hypotheses have been proposed up to the present.

As early as 1956, Coppen A et al. advanced a novel serotonin (5-HT) hypothesis on depression pathogenesis. In the central nervous system (CNS), 5-HT is an inhibitory neurotransmitter synthesized in the presynaptic neuron. 5-HT synthesis reduction, release reduction, or reuptake increase leads to a decrease of the 5-HT level in the synaptic cleft, and thus induces the onset of depression (Coppen, 1967). Based on this hypothesis, the selective serotonin reuptake inhibitors (SSRIs, such as fluoxetine and citalopram) have been developed and have been shown to improve symptoms in depression patients (Delgado, 2000).

In peripheral system, 5-HT as one of autacoids is synthesized and distributed in enterochromaffin cells, and stored in cell granules with ATP. Under the effect of stimulation factors, 5-HT is released from the granules, diffused into the blood, and absorbed and stored by platelets, accounting for about 8% of the total 5-HT in the body.

In this context, the correlation between 5-HT synaptic level and peripheral 5-HT level has become a curious concern of pharmacologists. Could the peripheral 5-HT level reflect the synaptic 5-HT level to some extent? Could the peripheral 5-HT level be used to diagnosis, to assess treatment efficacy of antidepressants? Many scientists have been efforted to better clarify the association, however, the efforts have been greatly hindered by the difficulty of direct measuring 5-HT level in patients’ synaptic cleft. Despite all this, researchers have conducted numerous studies on 5-HT level in plasma, serum and platelets in the diagnosis and treatment of depression, trying to confirm the possibility of the peripheral 5-HT level as a biomarker for depression diagnosis and treatment.

Unfortunately, as literature review found, no consistent evidence has been presented yet to clarify the relationship between peripheral 5-HT level and depression development. In this article, we reviewed the current evidence with the hope of providing ideas for the follow-up research on the relationship between 5-HT level and depression diagnosis and treatment.

## Methods

**FIGURE 1:**
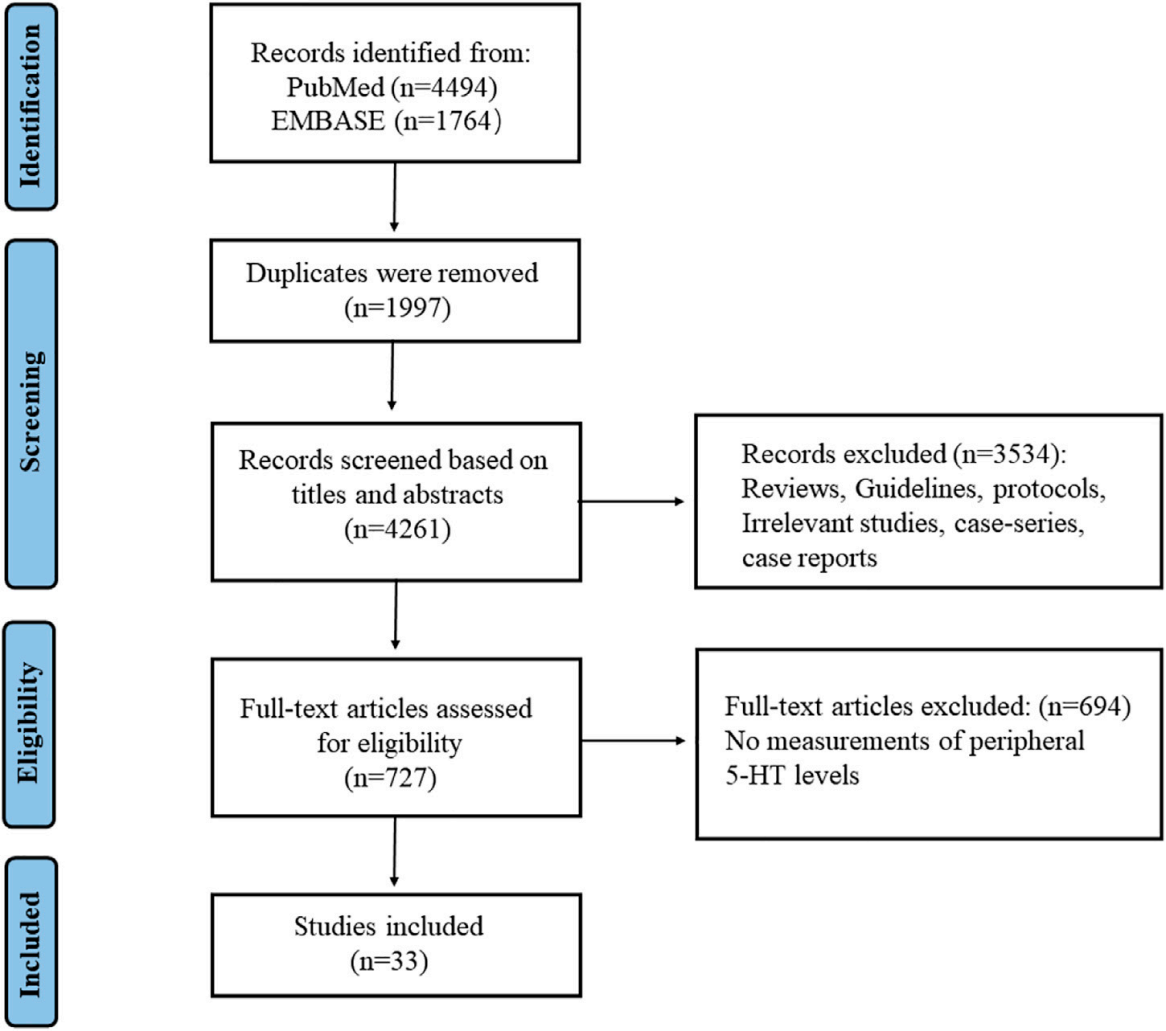
The search process and the screening of the articles for identifying the eligible studies.

PubMed and EMBASE were searched to collect relevant publications on peripheral 5-HT level and depression. The search formula of PubMed was (“Depression” [MeSH Terms] OR “Depress*” [tiab] OR “chronic mild stress” [tiab]) AND (“Serotonin/blood” [Mesh] OR “Serotonin” [Tiab] OR 5-HT [Tiab] OR “hydroxytryptamine” [Tiab]) AND (“peripheral” [tiab] OR “serum” [MeSH Terms] OR “serum” [tiab] OR “plasma” [MeSH Terms] OR “plasma” [tiab] OR “blood platelets” [MeSH Terms] OR “platelet*” [tiab] OR “blood” [tiab]) AND 1957:2022 [pdat]. And the search formula of EMBASE was (“depression”/mj OR “depress*”:ab,ti) AND (“serotonin”/mj OR “5-ht”:ab, ti OR “serotonin”:ab, ti OR “5-hydroxytryptamine”:ab,ti) AND (“serum”/mj OR “plasma”/mj OR “thrombocyte”/mj OR “serum”:ab, ti OR “plasma”:ab, ti OR “thrombocyte”:ab, ti OR “platelet”:ab,ti) AND ([animals]/lim OR [randomized controlled trial]/lim OR “controlled clinical trial”/de) AND [<1957-2022]/py. The detailed processes were shown in Figure 1.

### Depression clinical scale and behavior assessment in animal model

Briefly, the MADRS, HAMD, HDRS and HAMA are the most commonly used scales for assessing depression symptoms and for assessing antidepressants efficacy in clinical practice (Zimmerman et al., 2013; Obeid et al., 2018; Paketci, 2021). The depression score criteria are respectively as follows. The MADRS offers scores on the following scale: <12 (normal/remission), 12 to 22 (mild depression), 22 to 30 (moderate depression), 30 to 35 (major depression), >35 (extreme depression). The HAMD has two types of scoring: the HAMD-17 and the HAMD-24. The HAMD-17 is scored on a scale of 7–17 (probably depressed), 18 to 24 (depressed) and >24 (severely depressed); the HAMD-24 is scored on a scale of 9–20 (probably depressed), 21 to 35 (depressed) and >35 (severely depressed). The HDRS is rated on a scale of 0 (normal), 1 (mild depression), 2 (moderate depression), 3 (major depression) and 4 (very severe depression). The HAMA is scored on a scale as follows: <7 (no anxiety symptoms), 7 to 14 (possible anxiety symptoms), 14 to 21 (anxiety symptoms), 21 to 29 (significant anxiety symptoms), and ≥29 (severe anxiety symptoms).

Lots of evidences found that the negative life events are highly precipitating factors in developing depression. Chronic stress is the primary trigger for depression. An optimal animal model should replicate the signs, symptoms and behavioral manifestations of the disease observed in the clinic. Therefore, many chronic stress-induced depressive-like behavior animal model has been developed by using a range of mild, variable and random stimulus stressors on rats/mice. Among them CUMS (chronic unpredictable mild stress) animal model was a representative which was first designed in rats by Katz (Katz, 1982), and later improved by Papp and Willner (Papp et al., 1991; Willner et al., 1992). Besides, the chronic mild stress model of mice were first using by Monleon et al. (1995). Because of its effectiveness in perfectly simulating the clinical symptoms of depression to a large extent, this model is now the most widely used and appropriate animal model in depression research (Hao et al., 2019). A series of behavioral tests are widely used to assess depression severity, including sucrose preference test (SPT), tail suspension test (TST), forced swimming test (FST) and open field test (OFT) and so on (Hao et al., 2019). There is no recognized and objective biomarker for depression diagnosis in animal experiments.

### 5-HT level in various peripheral tissues and possible relevance with that in CNS

5-HT level in the cerebrospinal fluid (CSF) has been measured and documented its association with depression severity in patients with MDD (Hou et al., 2006). However, since it is not easy to obtain CSF sample from patients as well as brain tissue biopsy, few work directly measures the 5-HT level in the CNS up to date. The peripheral serum, plasma and platelet in circulation system therefore become accessible sample to test 5-HT level.

Various peripheral samples display different 5-HT level. For rough estimation, the 5-HT in serum equals to 5-HT in plasma and 5-HT in platelet (Lee et al., 2014; Choi et al., 2020), it is unclear that which sample of 5-HT is more related to the degree of depression. Based on results in 1,094 outpatients with depression, Choi et al. (2021) believed that serum 5-HT level could be used as a biomarker of antidepressants response . However, Mück-Seler et al. (2002) suggested that platelet 5-HT level could predict the therapeutic effect of paroxetine in depression patients. Bianchi et al. (2002) furthermore considered that platelet 5-HT might reflect 5-HT level in neuron in CNS, while plasma 5-HT reflect the 5-HT in synaptic cleft in experimental animal. The alterations of peripheral 5-HT level might have a ripple effect on 5-HT levels in the CNS (Bordukalo-Niksic et al., 2010). In fact, as the inhibition of platelet 5-HT uptake by antidepressants is highly consistent with the inhibition of synaptic 5-HT uptake (Da Prada et al., 1988), it is reasonable to consider that possibility of peripheral serum, plasma or platelet 5-HT to be used as biomarker of depression (Read et al., 2017).

### Inconclusive evidence of peripheral 5-HT level with depression/antidepressant treatment

In terms of group-comparison results, peripheral 5-HT level in depression patients demonstrated different outcome compared to normal healthy counterparts (shown in Table 1). Four studies showed that peripheral 5-HT level in depression patients were higher than normal subjects, one study reported peripheral 5-HT level in depression patients were lower than normal subjects, meanwhile two studies showed no significant difference.

**TABLE 1:** Periphery 5-HT levels in depressive patients compared to normal subjects.

Sample	5-HT results	References
Whole blood	Patients with current depression had higher whole blood 5-HT level than those without current depression.	[(Wulsin et al., 2009)]
Plasma	The 5-HT levels of plasma in the depressive patients were higher than that in healthy subjects, although the difference was not statistically significant.	[(Franke et al., 2000)]
Serum	The 5-HT level was significantly higher in depressive patients compared to control group.	[(Tyano et al., 2006)]
Platelets	Children with a mood disorder had significantly higher platelet 5-HT level than those without a mood disorder.	[(Pfeffer et al., 1998)]
Platelets-poor plasma platelet	The 5-HT level in platelets and platelets-poor plasma in MDD groups were lower than that in healthy group.	[(Zhang et al., 2014)]
Whole blood platelet	The 5-HT level in whole blood or platelet in patient with major depression did not differ significantly compared to that in healthy controls.	[(Mann et al., 1992)]
	However, platelet 5-HT level was correlated negatively with severity of depression in the patient group.	
Serum	The serum 5-HT level was not different between the healthy controls and MDD group.	[(Greaney et al., 2020)]

Fifteen papers on the relevance between peripheral 5-HT level and the drug treatment response in depression patients were retrieved (Table 2). The samples were whole blood, serum, plasma and platelets from depression patients. In terms of self-comparison results, five researches reported an increase in peripheral 5-HT levels after drug treatment. Nine researches reported a diametrically opposite result. The remaining one work reported no definite changes in peripheral 5-HT levels after drug treatment.

**TABLE 2:** Changes of peripheral 5-HT level in patients with depression after drug treatment.

Drugs	Sample	5-HT level change after treatment	References
Ketamine	Serum	Significantly increased.	[(Liu et al., 2021)]
Ketamine	Serum	Significantly increased as compared to the control group.	[(Wang et al., 2020a)]
SSRIs	Serum	Higher as compared to the control group.	[(Liu et al., 2015)]
Fluoxetine	Serum	Increased.	[(Zhou et al., 2015)]
Mirtazapine	White blood platelets	Higher as compared to the control group;	[(Schins et al., 2004)]
		Higher as compared to the control group.	
Fluoxetine	Serum	Significantly decreased.	[(Alvarez et al., 1999b)]
	Plasma	Significantly decreased	
Fluoxetine and Clomipramin	Plasma	Decreased.	[(Alvarez et al., 1999a)]
	Platelet	Decreased.	
Escitalopram	Platelet	Decreased.	[(Dvojkovic et al., 2021)]
Vortioxetine			
Citalopram	Platelet	Decreased.	[(Bismuth-Evenzal et al., 2012)]
Escitalopram			
Fluoxetine	Plasma	Increased.	[(Castrogiovanni et al., 2003)]
	Platelet	Decreased.	
Paroxetine	Whole blood	Decreased.	[(Figueras et al., 1999)]
	Plasma	Decreased.	
Sertraline	Platelet	Decreased.	[(Pivac et al., 2003)]
Escitalopram	Platelet	Decreased.	[(Zhuang et al., 2018)]
Fluoxetine	Platelet	Lower.	[(Li et al., 2015)]
Paroxetine			
Sertraline			
Citalopram			
SSRIs	Serum	Increased in half-patients, and decreased in half-patients.	[(Choi et al., 2021)]
Non-SSRIs		Increased in half-patients, and decreased in half-patients.	

### Inconclusive evidence of peripheral 5-HT level in CUMS-induced depressive-like behavior animal model

Since it is relatively easy to obtain sample from animal brain tissue, many studies were conducted to explore the association between central 5-HT level and depressive behavioral performance in animals. Most studies seem to support the 5-HT hypothesis. The 5-HT level in the hippocampus, cortex and prefrontal cortex (PFC) of depression-phenotype mice or rats were significantly reduced compared to the normal groups, and after antidepressant treatment, the 5-HT levels were increased and the animals’ depressive behaviors were significantly improved. Of course, there was report of contrary results. One study showed that the 5-HT levels of depression-phenotype rats in hippocampus were significantly increased compared to the normal group, while decreased after treating with citalopram, a SSRI (Li et al., 2015).

As to peripheral 5-HT level, elevenpapers on the relevance between peripheral 5-HT level and the antidepressant treatment response in depression-phenotype mice or rats were retrieved (Table 3). Eight experiments showed that the peripheral 5-HT level in depression-phenotype mice or rats were lower than normal ones, and after antidepressant treatments, the 5-HT levels were increased combined with improvements in depressive-like behavior. Two studies showed that the peripheral 5-HT levels were decreased after antidepressant treatments. The remaining one study reported no significant differences in peripheral 5-HT levels after antidepressant treatments, although the depressive-like behavior in animal were improved. These different or contradictory results may be explained by different animal species, modelling methods, drugs (including doses and period), sample and sampling methods, measuring methods, and so on.

**TABLE 3:** Evidence of peripheral 5-HT level in CUMS-induced depressive-like behavior animal model.

Animals	Drug	Duration of treatment	5-HT level change after CUMS	5-HT level change after treatment	References
Male SPF C57BL/6 mice	Fluoxetine	28 days	Decreased.	Increased.	[(Chen et al., 2021a)]
Male SPF Wistar rats	Taurine	28 days	Decreased.	Increased.	[(Wu et al., 2017)]
Male C57BL/6 mice	Fluoxetine	42 days	No changed.	Increased.	[(Cheng et al., 2021)]
Male athymic nude mice (NCr-nu)	Fluoxetine	21 days	Decreased.	Increased.	[(Zhang et al., 2020)]
Female C57BL/6 mice	Fluoxetine	14 days	Decreased.	Increased.	[(Yang et al., 2021)]
Male CD-1 mice	Sesame	42 days	Decreased.	Increased.	[(Zhao et al., 2019)]
Male and female SD rats	Trimetazid-ine	28 days	Decreased.	Increased.	[(Liu et al., 2018a)]
Male C57BL/6 mice	Fluoxetine	35 days	Decreased.	Increased.	[(Oh et al., 2020)]
Male SD rats	Citalopram	42 days	No differences.	Decreased.	[(Li et al., 2015)]
Male SD rats	Fluoxetine	28 days	Increased.	Decreased.	[(Li et al., 2013)]
Male Wistar rats	Prebiotics (FOS/GOS)	28 days	No changed.	No changed.	[(Li et al., 2019)]
	Probiotics (B.l and L.r)				

### Peripheral 5-HT level could not be used as a biomarker for depression diagnosis and treatment

Many SSRIs based on 5-HT hypothesis have made a significant contribution to the treatments of depressive disorders. 5-HT level has been used to guide clinical decision on whether to use or continue to use, or even discontinue antidepressants (Maund et al., 2019). Here 5-HT level should be 5-HT in synaptic cleft rather than in peripheral system. Just due to the difficulty in sampling from CNS, peripheral sample has to be adopted to replace in measuring central 5-HT level (van Buel et al., 2019). From the evidence listed above, unfortunately, it does not work. Peripheral 5-HT level could not be used as a biomarker both for depression diagnosis and for antidepressant efficacy evaluation.

The inconsistent results suggested that the onset of depressive disorders might not be solely induced by a deficiency of 5-HT. It might be related to many other factors such as receptors on dendrites, the receptive apparatus of nerve cells, various subtypes of 5-HT receptors for various antidepressant drugs (Yohn et al., 2017). A combination of factors such as dosing cycles, patient’s age, race, gender, environment and dietary habits might have contribution to this inconsistence.

## Conclusion

The peripheral 5-HT levels showed inconsistent results before and after antidepressant treatments both in clinical trials and animal experiments. In summary, peripheral 5-HT level could not be used as a biomarker both for depression diagnosis and for antidepressant efficacy evaluation..

## Significance statement

This research determined that peripheral 5-HT level could not be used as a biomarker both for depression diagnosis and for antidepressant efficacy evaluation, and plasma 5-HT level might be a possible biomarker to demonstrate its association with depression. However, our samples so small that perhaps with larger samples, the conclusions—especially those relating to animal data—might change. This study will help researchers pay more attention to the relationship between plasma 5-HT and depression. In future, more and more work should be efforted to achieve comparability of data.
