# Corrigendum: Could peripheral 5-HT level be used as a biomarker for depression diagnosis and treatment? A narrative minireview

**DOI:** 10.3389/fphar.2023.1318433

**Published:** 2023-11-09

**Authors:** Canye Li, Qiming Cai, Zuanjun Su, Zhicong Chen, Jinming Cao, Feng Xu

**Affiliations:** ^1^ Fengxian Hospital, Southern Medical University, Shanghai, China; ^2^ Sixth People’s Hospital South Campus, Shanghai Jiaotong University, Shanghai, China

**Keywords:** 5-HT, peripheral, depression, biomarker, plasma, platelet

In the published article, there was an error in **Affiliation** 1. Instead of “School of Pharmaceutical Sciences, Southern Medical University, Guangzhou, China.” it should be “Fengxian Hospital, Southern Medical University, Shanghai, China”.

The authors apologize for this error and state that this does not change the scientific conclusions of the article in any way. The original article has been updated.

